# Propensity to sell stocks in an artificial stock market

**DOI:** 10.1371/journal.pone.0215685

**Published:** 2019-04-25

**Authors:** Wlademir Prates, Newton Da Costa, Manuel Rocha Armada, Sergio Da Silva

**Affiliations:** 1 Department of Business Administration, Federal University of Santa Catarina, Florianopolis, Brazil; 2 Department of Economics, Federal University of Santa Catarina, Florianopolis, Brazil; 3 Business School, Pontifical Catholic University of Parana, Curitiba, Brazil; 4 School of Economics and Management, University of Minho, Braga, Portugal; BeiHang University School of Economics and Management, CHINA

## Abstract

This experimental study of an artificial stock market investigates what explains the propensity to sell stocks and thus the disposition effect. It is a framed field experiment that follows the steps of a previous observational study of investor behavior in the Finnish stock market. Our experimental approach has an edge over the observational study in that it can control extraneous variables and two or more groups can be compared. We consider in particular the groups of amateur students and professional investors because it is well established in the literature that the disposition effect is less pronounced in professionals. The disposition effect was measured by both the traditional metric and a broader one that properly considers return intervals. A full logit model with control variables was employed in the latter case. As a result, we replicate for the broader definition what already has been found for the traditional measure: that investor experience dampens the disposition effect. Trades with positive returns exhibited higher propensity to sell than trades with negative returns. For the overall sample of participants, we find the disposition effect cannot be explained by prospect theory, but we cast doubt on this stance from partitions of data from amateurs and professionals.

## Introduction

Investors usually take longer to sell stocks that have lost value relative to their purchase price than they take to sell those that have increased in value since acquisition. The financial literature refers to this behavior as the disposition effect. Under the disposition effect, investors tend to sell more stocks with positive returns than those with negative returns. The disposition effect is one of the most studied behavioral biases in finance [1]. Many explanations have been proposed but none is established [2] [3]. One particular aspect of interest in our study of the phenomenon is investor experience. Professionals are more likely to escape the disposition effect, a result shown both behaviorally [4, 5] and with neural data [2].

The type of experiment considered in this study can be labeled as a framed field experiment [6]. A computer program is designed to simulate a user interface homebroker, which is a tool commonly used by investors, and the participants in the experiments are amateur students and professional investors. These two groups are considered because the distinction between amateurs and professionals matters for the disposition effect, as observed. In this experiment, we consider the insights previously suggested in an observational study of investor behavior in the Finnish stock market [3]. The advantages of our artificial stock market approach over the observational study include: our experiment can give evidence that the treatment actually causes the response in that it controls extraneous variables; it can compare two or more groups; it is cheaper, shorter, simpler to implement, easier to replicate, and can consider more than one explanatory variable.

Of note, we build an artificial stock market merely as an input for our experimental study that aims to have an edge over the observational study we use as a reference. This means our artificial market is built only as a way to make viable a framed field experiment. The stock market is artificial in the sense that a computer program is designed to simulate a real-world user interface homebroker and participants make decisions using a software we built to simulate the market. Therefore, what we call an artificial stock market is not to be confused with an agent-based modeling, where the artificial market is full-blown and agents differ in many ways, not just in their information, but in their ability to process information, their attitudes toward risk, and in many other dimensions, including trading experience and cognitive ability [7]. However, like our artificial market, the agent-based model challenges the traditional fully rational homogeneous perspective and offers a new behavioral approach where participants are heterogeneous and have bounded rationality [8]. For comprehensive, recent reviews of agent-based modeling in finance, see Refs. [8–10].

Our analytical method employs a logistic regression model to estimate the probability of a sale based on the stock return in a specific time period. This means our study focuses on one individual investor’s propensity to sell a stock. The dependent variable in the model is the decision to sell compared to the decision to hold the stock in the portfolio, and it is expressed as a binary value. The independent variables are indicators (also in binary form) of gains and losses over a range of different return intervals, expressed as percentages of gains and losses accruing from the original purchase price. The regressions also include a set of control variables.

The rest of the paper is organized as follows. Section 2 presents the materials and methods, Section 3 shows the results and Section 4 concludes this report.

## Materials and methods

### Metrics

This study measures the disposition effect by both the original metric suggested by Odean [11] and the extension proposed by Grinblatt & Keloharju [12]. The latter is also employed by Kaustia [3], the benchmark study to which ours is compared. The Odean’s metric is used to calculate the disposition coefficient (DC) for each participant by assessing the proportion of gains realized (PGR) and the proportion of losses realized (PLR). If PGR > PLR, then an individual participant exhibits the disposition effect. Of note, DC = PGR–PLR. Kaustia’s metric, which happens to be also that of Grinblatt & Keloharju, is used to estimate the probability of selling a stock at a given level of return through logistic regressions, in particular a logit model. Following Kaustia, we label such an estimated probability as the propensity to sell. Here, one individual behaves in line with the predictions of the disposition effect literature if she displays greater propensity to sell for intervals of positive returns than for intervals of negative returns.

In either metric, one has to first set a reference point to calculate the purchase price of one stock in order to eventually calculate the amplitude of the returns from each trade. Sale prices higher than the reference point represent gains (positive returns), while sale prices below the reference point refer to losses (negative returns). In this study, the reference point chosen to calculate the purchase price is the average price of an acquisition, as in Odean [11]. In the presence of either extra purchases of a stock already held or of partial sales of a stock held, the first-in-first-out criterion [3] is applied to recalculate the average price of acquisition.

We also follow Kaustia [3] and analyze the data using a logistic regression model, where the dependent variable takes on the value of 1 when a stock is sold, or 0 when the stock is kept in the portfolio. The independent variables are a set of binary variables that classify the returns from a sell (gains realized) and the potential returns that are not realized when a stock is held in the portfolio (gains unrealized) at various percentage levels. The aim is to identify those return intervals that have the greatest power to explain one participant’s decision to sell or hold a stock.

### Odean’s metric

Here we detail Odean’s metric [11] following our own presentation published elsewhere [2]. In a given period of time, each stock is assigned to one of four categories: 1) as a gain realized, whenever a stock is sold at a price that is higher than the average purchase price; 2) as a loss realized, whenever it is sold at a price that is lower than the average purchase price; 3) as a paper gain, whenever its price is higher than its average purchase price, but the stock is not sold during that period; and 4), as a paper loss, whenever its price is lower than its average purchase price, but the stock is not sold during the period. The proportion of gains realized (PGR) computes the number of gains realized as a fraction of the number of gains that could have been realized. It is then

$$\mathrm{PGR}=\frac{\mathrm{total}\;\mathrm{number}\;\mathrm{of}\;\mathrm{gains}\;\mathrm{realized}}{\mathrm{total}\;\mathrm{number}\;\mathrm{of}\;\mathrm{gains}\;\mathrm{realized}+\mathrm{total}\;\mathrm{number}\;\mathrm{of}\;\mathrm{paper}\;\mathrm{gains}}.$$ (1)

And the proportion of losses realized (PLR) is

$$\mathrm{PLR}=\frac{\mathrm{total}\;\mathrm{number}\;\mathrm{of}\;\mathrm{losses}\;\mathrm{realized}}{\mathrm{total}\;\mathrm{number}\;\mathrm{of}\;\mathrm{losses}\;\mathrm{realized}+\mathrm{total}\;\mathrm{number}\;\mathrm{of}\;\mathrm{paper}\;\mathrm{losses}}.$$ (2)

As observed, the disposition effect occurs if PGR > PLR, in which case the disposition coefficient (DC) is positive.

Despite its virtues, one downside of Odean’s metric is that a gain realized is computed independently of its amplitude. For example, a gain realized of 5 percent has the same impact on DC as a gain realized of 50 percent. Moreover, since DC is the result of two ratios, it is nonlinear [5]. Though these drawbacks do not invalidate Odean’s metric, it is still of interest to consider Kaustia’s metric and investigate how investors behave in response to changing levels of possible returns, in both the domain of gains (positive returns) and the domain of losses (negative returns). This leads to the investigation of which return intervals are more likely to increase one investor’s propensity to sell a stock in her portfolio.

### Propensity to sell

It is thus relevant to study the disposition effect after the classification of returns into intervals [3, 12]. This leads to Kaustia’s metric. To determine which return interval increases the propensity to sell, discrete choice regression models–such as those from the logistic regression family–can be used. These models can estimate the probability that one investor will sell a stock in the presence of variations in the explanatory variables of the model. In particular, logit models can be successfully employed [3, 12].

### Logistic regression

Logistic regression models are appropriate for dealing with qualitative data such as ours. They model situations in which the dependent variable *Y* is binary (in that it takes on the values of 0 or 1) and the value that is sought is the probability *π* that variable *Y* equals 1, given a specific value for a variable *X*, which could be either binary or categorical [13]. Thus,

π=Pr(Y=1|X=x). (3)

The relationship between *π* and *X* can be modeled as a logistic response function:

$$\pi =\mathrm{Pr}(Y=1|X=x)=\frac{e^{\beta_{0}+\beta_{1}x_{1}+\cdots +\beta_{p}x_{p}+\varepsilon }}{1+e^{\beta_{0}+\beta_{1}x_{1}+\cdots +\beta_{p}x_{p}+\varepsilon }},$$ (4)

which is nonlinear. However, it can be made linear by taking its natural logs on both sides, that is,

$$\mathrm{logit}\pi =\ln\frac{\pi }{1-\pi }=\beta_{0}+\beta_{1}x_{1}+\cdots +\beta_{p}x_{p}+\varepsilon ,$$ (5)

where $\ln\frac{\pi }{1-\pi }$ has an interval that extends from −∞ to +∞, a property that meets our needs in here. The logit model is linear in the parameters, which are estimated by maximum likelihood; the coefficients *β*s are the logarithms of odds ratios [13]. After estimation, the probabilities of each value of *x*_*p*_ are obtained.

As observed, in our model the dependent variable *Y* is binary; *Y* = 0 whenever one participant does not sell a stock in a given period, and *Y* = 1 when a stock is sold. Holding a stock (*Y* = 0) means a gain or loss unrealized, while selling it (*Y* = 1) means a gain or loss realized.

Here, such gains and losses realized and unrealized are associated with their corresponding return intervals. We set each of these intervals at 10 percent. Sales at the same price as purchases (that is, trades with a return of 0 percent) are dismissed, since the disposition effect refers to situations of gains and losses only. Each 10 percent return interval is represented by the independent binary variable *x*_*p*_; *x*_*p*_ = 1 for a sale in a given period for interval *p*, and *x*_*p*_ = 0 for no sale.

The purpose of this logistic regression model is to identify which return interval, whether positive or negative, best explains selling behavior (*Y* = 1). If the disposition effect is at work, we expect participants in our experiment to exhibit greater propensity to sell a stock that offers positive returns. In contrast, we expect the participants to hold a stock in their portfolios in the presence of negative returns.

### Data collection and experiment design

We recruited 21 professional investors (2 females, age range 20 to 48, mean age 26.4) and 46 university students (18 females, age range 19 to 32, mean age 22.4) (*n* = 67). The professionals were financial traders from firms located in the Florianopolis area in southern Brazil. We picked professionals with at least two years of experience in stock trading. Nine professionals reported more than five years of experience, while 12 reported from two to five years. The students came from the Federal University of Santa Catarina, also located in Florianopolis. They were undergraduates enrolled in economics, accounting or management. We chose those students who had already taken the course Capital Markets. The experiment with students was conducted during two sessions run in the second term of 2017. One participant in the subsample of students had more information about the stock market than an individual picked at random from a larger population that includes non-students. This means we took a conservative stance while sampling unexperienced investors, because this group was recruited from undergraduates with basic notions of how the stock market works. The research received approval from the Ethical Committee for Research on Human Beings of the Federal University of Santa Catarina (case number: 1.744.242; date of approval: September 26, 2016).

The experiments were conducted using a software that simulates the stock market, called SimulaBolsa. The market is exogenous to one participant in that her trading does not impact a stock price. The experiments had an average session duration of 90 minutes for both groups of students and professionals. The experimental sessions for the students took place in the university’s financial markets laboratory, and participants were assigned to individual desktops with no communication between each other. The experimenter (W.P.) provided instructions at the beginning of each session. As for the professionals, they were approached by the experimenter on a one-to-one basis at their workplaces. Otherwise, they could not take part in the experiment.

Table 1 shows some descriptive statistics of the participants and the number of trades made in the experiment. As can be seen, most participants were young males and the students executed more buy and sell transactions than the professionals did.

**Table 1:** Descriptive statistics for the sample.

	Gender		Age			Sell and buy transactions			
	Male	Female	Mean	Minimum	Maximum	Mean	Minimum	Maximum	Total
Students	28	18	22.4	19	32	72	15	203	3,315
Professionals	19	2	26.4	20	48	57	15	194	1,198
Total	47	20	23.6	19	48	67	15	203	4,513

### Simulation

The computer program that simulated the stock market generated an individual report for all the decisions made by the participants throughout the simulation period. The output could thus allow one to get informed about the variables, such as the number of stocks bought and sold during each period and an individual portfolio composition at the end of a period. The program was fed with actual data for stock prices taken from the Sao Paulo stock exchange for the five-year period from January 1997 to December 2001. The program also included indicators based on fundamental analysis taken from Economatica. However, the participants were not informed about that, and the companies’ real names were replaced with fictitious ones. The stock prices were deflated by the Brazilian GDP deflator and corrected for dividends. Then, the prices were normalized so that each stock cost one Brazilian real (R$ 1) at the beginning of the experiments. Because prices (purchase prices equaling selling prices) were fixed by the simulator, the whole stock market was exogenous to one participant. Each participant was then considered as a small trader and their actions did not influence prices. Participants were then asked to manage the portfolio over 20 periods using the program, and their buying and selling decisions for 28 different stocks had to be made at the beginning of each period. Decisions were to be reached within a three minute time limit. After this limit, a simulation screen switched for the next one. The participants could eventually compare their decisions during the experiment with the actual stock prices announced by the program. More details on this program can be found elsewhere [4].

The output generated by the program thus consisted of a list of transactions made by the participants. This output made it possible to compare the value of one participant’s final portfolio with the initial sum she invested and then calculate the total return for the entire simulation. We developed another algorithm in Matlab (available upon request) to calculate both the returns on sell transactions period by period and the paper returns that were available when one participant decided not to sell a stock held in her portfolio. We then reconstructed each participant’s portfolio at the end of each period, making it possible to calculate both the returns realized (when a stock was sold) and the returns unrealized (when a stock was held).

## Results

### The disposition coefficient and the proportion of gains and losses realized

Table 2 shows the disposition coefficients for the whole sample and subsamples that consider investor experience (amateurs and professionals), gender (males and females) and age (age interval [19, 22 [and age interval [22, 48]). We arbitrarily divide the age interval into two groups by 22 because this is the mean age of the students. As can be seen, all the groups exhibited the disposition effect according to Odean’s metric (DC > 0). The *Z* statistic, which is the result of tests of the difference between proportions, where H1: PGR > PLR shows that DC was statistically greater than zero at the significance level of 1 percent, apart from the group of professionals, where DC > 0 was significant at 10 percent.

**Table 2:** Disposition coefficients for distinct groups.

	Whole sample	Students	Professionals	Males	Females	Age interval [19, 22]	Age interval [22, 48]
*n*	67	46	21	47	20	20	47
PGR	.225	.262	.152	.210	.260	.318	.190
PLR	.115	.109	.129	.127	.091	.106	.119
DC	.110	.154	.023	.083	.169	.212	.071
*SE*	.008	.01	.014	.01	.015	.016	.009
*Z* statistic	13.56***	15.20***	1.68*	8.44***	11.68***	13.27***	7.24***

*significant at 10 percent

***significant at 1 percent

The proportion of losses realized (PLR) did not vary a great deal across the groups, ranging from .091 to .129. However, the proportion of gains realized (PGR) showed expressive variation across the groups, ranging from .152 (professionals) to .318 (younger participants). This volatile PGR was the major factor responsible for the differences in DC across the groups.

Table 3 shows the disposition effect for the 10 percent return intervals. We considered negative return intervals for both losses unrealized (*Y* = 0) and losses realized (*Y* = 1), and then computed PLR. We also considered the situations where the participants held a stock (*Y* = 0) or sold it (*Y* = 1) thus obtaining positive returns, and then computed PGR. As can be seen, PGR > PLR for all the intervals (significant at 1 percent, apart from the interval ]-60, -50]), thus suggesting the presence of the disposition effect.

**Table 3:** The disposition effect for return intervals.

Negative intervals, %	*Y* = 0	*Y* = 1	PLR	Positive intervals, %	*Y* = 0	*Y* = 1	PGR	PGR > PLR *Z* statistic
[-100, -90]	121	1	.008	[90, 100]	109	40	.268	6.99***
[-90, -80]	130	10	.071	[80, 90]	94	22	.190	2.79***
[-80, -70]	89	11	.110	[70, 80]	92	30	.246	2.72***
[-70, -60]	148	12	.075	[60, 70]	149	40	.212	3.77***
[-60, -50]	298	54	.153	[50, 60]	241	55	.186	1.09
[-50, -40]	397	31	.072	[40, 50]	231	76	.248	6.34***
[-40, -30]	490	54	.099	[30, 40]	345	118	.255	6.49***
[-30, -20]	593	90	.132	[20, 30]	451	108	.193	2.91***
[-20, -10]	733	106	.126	[10, 20]	646	168	.206	4.39***
[-10, 0]	769	125	.140	[0, 10]	671	171	.203	3.50***

***significant at 1 percent

Participants made zero returns in some trades where the sale price matched the purchase price and thus PGR and PLR could not be computed. Such data were dismissed from Table 3. There were 207 such zero returns unrealized and 34 zero returns realized. Moreover, arithmetic (non-logarithmic) returns were considered and thus there were no returns lower than −100 percent, but there were many returns greater than +100 percent. This asymmetry explains why Table 3 shows no positive returns above 100 percent, in which case the *Z* statistic could not be computed due to the absence of returns for the corresponding negative interval.

The program output data provided observations for all the stocks in all the periods. However, observations were null in those cases where a participant did not hold any stocks. Such null observations were also dismissed. As a result, we ended up with a total number of observations of 277,695, arranged in a matrix with 8,415 lines and 33 variables, including the binary ones for the 10 percent return intervals and also non-binary and categorical variables.

### Variables explaining selling behavior

Table 4 shows the results of a full logit model that further considers the control variables *c* in addition to the return interval variables *x*, that is,

$$\mathrm{logit}\pi =\beta_{0}+\beta_{1}x_{1}+\cdots +\beta_{p}x_{p}+\beta_{p+1}c_{1}+\cdots +\beta_{p+j}c_{j}+\varepsilon ,$$ (6)

where *π* is the probability of selling (*Y* = 1), the *β*s are the coefficients, and *ε* is the error term.

**Table 4:** Variables explaining selling behavior.

Panel A: return interval variables					
Negative intervals, %	Coefficients	*SE*	Positive intervals, %	Coefficients	*SE*
Constant	-1.587***	.323	[100, ∞]	.460**	.233
[-100, -90]	-2.265**	1.044	[90, 100]	.995***	.295
[-90, -80]	-.693*	.417	[80, 90]	.577*	.339
[-80, -70]	-0.321	.410	[70, 80]	.955***	.316
[-70, -60]	-.723*	.376	[60, 70]	.595**	.286
[-60, -50]	-.047	.271	[50, 60]	.397	.274
[-50, -40]	-.737***	.294	[40, 50]	.780***	.262
[-40, -30]	-.431*	.262	[30, 40]	.791***	.242
[-30, -20]	-.160	.246	[20, 30]	.514**	.244
[-20, -10]	-.195	.241	[10, 20]	.414*	.233
[-10, 0]	-.110	.234	[0, 10]	.477**	.228
Panel B: control variables					
Control variables	Coefficients	*SE*			
experience	.189**	.083			
gender	-.021	.067			
age [22, 48]	-.266***	.070			
trade [46, 65]	.336***	.093			
trade [65, 79]	.052	.095			
trade [79, ∞]	-.034	.094			
Panel C: robustness of the model					
Y = 0: *n* = 13,900, *Y* = 1: *n* = 4,395, Total: *n* = 18,295					
AIC: 7,455.6					
McFadden pseudo-R^2^: .061					
LR statistic: 477.27***					

*significant at 10 percent

**significant at 5 percent

***significant at 1 percent

The control variables *c* considered were experience, gender, age, number of trades, stock and period. “Experience” and “gender” were binary: 1 for professional and 0 for student, and 1 for male and 0 for female. “Age” was divided into two percentiles, and the lower age percentile was removed to avoid problems of multicollinearity. The total number of trades made by each participant was expressed in four quartiles, and the bottom quartile was dismissed to prevent multicollinearity and only used as a reference point for the interpretation of results. Variable “trade [79, ∞[,” for example, refers to the top quartile: 75 percent to 100 percent. “Stock” takes on the values from 1 to 28 to refer to each of the 28 stocks traded, and “period” contained 20 categories to reflect the 20 trading periods of the experiments.

Of note, the sum of all binary variables relative to return intervals is always equal to 1 because gains or losses realized and unrealized always offer returns that can be ascribed to one of the 10 percent intervals. To avoid falling into the “dummy variable trap,” which could cause incorrect interpretation of the results, the independent variable representing the trades that resulted in returns of 0 percent was removed from the set of explanatory variables and a constant was included in the model instead. Inclusion of such a constant has the effect that the removed variable can be considered as a reference for interpretation of the coefficients. The significance of the coefficients was evaluated by Wald tests.

Table 4 shows the negative returns that exhibited statistical significance were those for the intervals −30 percent to −50 percent, and for the intervals −80 percent to −100 percent. The interval ]−70, −60] was also statistically significant. As for the positive returns, only the interval [50, 60 [did not have a statistically significant impact on selling behavior.

It was impractical to show the large numbers of levels of the control variables “stock” and “period” in Table 4. However, from the 28 different stocks only four exhibited statistical significance at the 10 percent level. From the 20 periods, 12 showed significance at 10 percent, and the later periods (from 14 to 20) were most relevant to explain selling behavior.

### Propensity to sell

The full logit model can be used to assess the propensity to sell a stock. Propensity to sell is the probability estimated by the model that a sale will be executed (*Y* = 1) for a return interval *x*: Pr(*x* = 1). Table 5 shows that trades with positive returns exhibited higher propensity to sell than trades with negative returns. The probability of executing a sell transaction was greater if the sale realized a gain, and participants were reluctant to realize losses. This result provides evidence of the disposition effect according to Kaustia’s metric, and experimentally replicates that of the observational study of Kaustia [3]. Observe in Table 5 that the propensity to sell at a gain or a loss was relatively constant across both the negative and positive intervals, a finding that challenges the explanation for the disposition effect based on prospect theory. Prospect theory predicts the propensity to sell a stock will decrease as its price moves away from the purchase price in either direction, though the movement is asymmetric for gains and losses. This point was first observed by Kaustia [3] and here we experimentally give support for his claim.

**Table 5:** Propensity to sell for each return interval.

Return intervals	Coefficients	*SE*	Propensity to sell Pr(*x* = 1)	Lower limit	Upper limit
Constant	-1.797***	.197	-9.1200		
[-100, -90]	-2.691***	1.023	.0111	.0016	.0744
[-90, -80]	-.645*	.384	.0800	.0436	.1423
[-80, -70]	-.0218	.390	.1176	.0645	.2050
[-70, -60]	-.562	.361	.0863	.4970	.1459
[-60, -50]	.049	.253	.1483	.1132	.1918
[-50, -40]	-.748***	.278	.0727	.0507	.1033
[-40, -30]	-.328	.247	.1067	.0820	.1377
[-30, -20]	-.128	.232	.1273	.1030	.1563
[-20, -10]	-.124	.226	.1277	.1055	.1538
[-10, 0]	-.125	.225	.1276	.1059	.1529
[0, 10]	.467**	.217	.2091	.1810	.2404
[10, 20]	.528**	.217	.2194	.1905	.2513
[20, 30]	.478**	.228	.2109	.1760	.2506
[30, 40]	.827***	.227	.2748	.2329	.3211
[40, 50]	.762***	.244	.2621	.2111	.3204
[50, 60]	.416*	.254	.2008	.1552	.2558
[60, 70]	.552**	.270	.2235	.1671	.2923
[70, 80]	.821***	.294	.2736	.1973	.3660
[80, 90]	.436	.319	.2041	.1356	.2953
[90, 100]	.907***	.274	.2910	.2204	.3734
[100, ∞]	.502**	.210	.2149	.1924	.2392
*Y* = 0: *n* = 13,900, *Y* = 1: *n* = 4,395, Total: *n* = 18,295 AIC: 7,597 McFadden pseudo-R^2^: .029 LR statistic: 226,040***					

*significant at 10 percent

**significant at 5 percent

***significant at 1 percent

**Fig 1:**
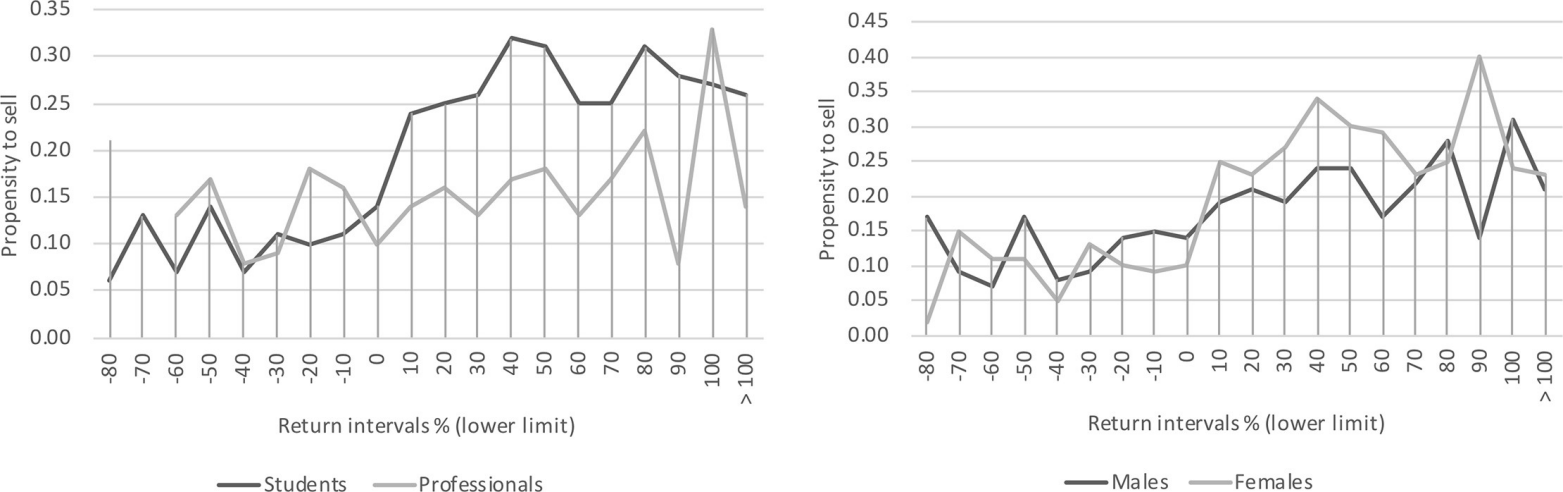
A. Students versus professionals: the propensity to sell stocks is not stable across the return intervals when particular groups are considered. B. Males versus females: the propensity to sell stocks is not stable across the return intervals when particular groups are considered.

Nevertheless, when we run the full logit model taking data from students, professionals, males and females separately, we cast doubt on the stability of the propensity to sell across the return intervals. Fig 1 shows that the propensity to sell remains constant across the negative return intervals, but increases across the positive ones. The only group for which this is not clear cut is that of professionals (Fig 1A). So, perhaps prospect theory could not explain the disposition effect for professional investors, supposedly the vast majority in the observational sample of the Finnish stock market used by Kaustia. However, this does not seem to be obvious for amateurs and other particular groups, as our experimental study suggests.

To quantify the disposition effect when return intervals are considered (Kaustia’s metric), we run our full logit model, this time to consider two binary explanatory variables: one for positive returns and the other for negative returns. Our objective is to assess whether the probability of executing a sale of a stock at a profit is higher than the probability of selling it at a loss. Further, we consider the result for the groups of students and professionals separately.

Table 6 shows the results where two dummies for return intervals were considered. The LR statistic was calculated to compare the model tested with a model restricted, with no explanatory variables. As can be seen, the amateurs (students) were 2.38 times more likely to execute a sale transaction to realize a gain than to execute one to realize a loss. The professionals showed a lesser inclination (1.17). This result is in line with the literature that investor experience dampens the disposition effect. However, such a result has been shown for Odean’s metric of the disposition effect [4, 5]. Here, we have extended it to the broader Kaustia’s metric of the disposition effect that properly considers return intervals.

**Table 6:** Propensity to sell for negative and positive return intervals, by group.

Probability	Overall	Students	Professionals
Pr(*Y* = 1 | return > 0)	.23	.26	.15
Pr(*Y* = 1 | return < 0)	.12	.11	.13
Pr(gain)/Pr(loss)	1.94	2.38	1.17
*n*	8,415	5,732	2,683
LR	177.09***	222.58***	2.71*
AIC	7,608	5,335	2,201
McFadden pseudo-R^2^	.02	.04	0

*significant at 10 percent

***significant at 1 percent

A final caveat is that the amplitudes of the intervals of gains and losses realized that are analyzed in this experimental study cannot be straightforwardly compared with those in Kaustia’s observational study [3]. Our study considered the percentage variation for each buy or sell period as equivalent to the percentage variation of one month of actual data from the stocks traded in the Sao Paulo stock exchange. In contrast, Kaustia considered daily data.

## Conclusion

This is a study of an artificial stock market that evaluates the propensity to sell stocks and thus the possible occurrence of the disposition effect. It is a framed field experiment that follows the steps of a previous observational study of investor behavior in the Finnish stock market [3]. The advantages of our artificial stock market approach over the observational study are: 1) our experiment can give evidence that the treatment actually causes the response in that it controls extraneous variables, and 2) two or more groups can be compared. We consider in particular the groups of amateur students and professional investors because it is well established in the literature that the disposition effect is less pronounced in professionals [4].

We detail the variables explaining the propensity to sell a stock and the emergence of the disposition effect in our experiments. The disposition effect was measured by both the traditional metric [11] and a broader one that properly considers return intervals [3]. A full logit model with control variables was employed in the latter case. As a result, we replicate for the broader definition what already has been found for the traditional measure: that investor experience dampens the disposition effect. Trades with positive returns exhibited higher propensity to sell than trades with negative returns.

For the overall sample of participants, we confirm Kaustia’s key finding [3] that the disposition effect cannot be explained by prospect theory. However, this result is not clear cut when groups of participants are considered. Indeed, we found the propensity to sell at a gain or at a loss was relatively constant across both negative and positive return intervals–a finding that challenges the explanation for the disposition effect based on prospect theory. Yet, when we considered data for groups of amateurs and professionals separately, we found that the propensity to sell stocks remained constant across the negative return intervals, but increased across the positive ones.
